# Response to: Where in the World (Spine) am I?

**DOI:** 10.1016/j.inpm.2022.100157

**Published:** 2022-11-04

**Authors:** Jatinder S. Gill, Steven P. Cohen, Thomas T. Simopoulos, Michael B. Furman, Salim M. Hayek, Koen Van Boxem, David J. Kennedy, W Michael Hooten, Vinil Shah, Milan P. Stojanovic

**Affiliations:** Harvard Medical School, Department of Anesthesiology, Critical Care and Pain Medicine, BIDMC, Boston, USA; Johns Hopkins Medicine, Baltimore, MD, USA; Harvard Medical School, Boston, MA, USA; Temple University, Philadelphia, PA, USA; Case Western Reserve University, Cleveland, OH, USA; Universitair Ziekenhuis Gent, Gent, Belgium; Vanderbilt University Medical Center, Nashville, TN, USA; Mayo Clinic College of Medicine and Science, Rochester, MN, USA; University of California, San Francisco, San Francisco, CA, USA; VA Boston Healthcare, Harvard Medical School, Boston, MA, USA

Dear Editor,

We want to thank Dr. Waring for drawing an analogy between radiofrequency cannula reporting on spinal imaging and GPS systems localizing a target on a map. Both the localizing modality (GPS or X-Ray) and the map must be precise for there to be accurate localization of target, as well as to preserve high fidelity in communications.

Medial branch radiofrequency ablation (RFA) is one of the more demanding procedures in Interventional Pain Medicine. Successful lesioning requires precise placement of the electrode tip very close to the target nerve and in the correct orientation using optimal imaging. Positioning the electrode tip sufficiently close to the nerve, and confirming correct placement are highly dependent on correct visualization of the target location. Lau et al. described the optimal position of an electrode in relation to a lumbar medial branch as “electrode lies hard up against the superior articular process, such that it lies medial to the lateral silhouette of the dorsal end of the superior articular process” [1]. The spatial relationship of the superior articular process (SAP) with the electrode tip can be distorted if the end plates are not aligned and the spinous process is not centered between the pedicles as in a true AP view, similarly this spatial relationship can also be distorted in the absence of true lateral view. In addition, parallax error, where the target is not close to the beam center, can also distort the image.

We assume that Dr. Waring is pointing to Fig ​6 in our article whereby the superior L3 end plate margins are not aligned as they are at L4 [2]. We agree that a true AP should be obtained with the end plates aligned for true localization of the electrode tip in relation to the SAP, and a true lateral should be obtained as well. Nevertheless, the ideal areas for lesioning as noted in our article and based upon the publication by Lau et al. [1] and the Spine Intervention Society Guidelines [3] remain unchanged, along with the additional caveat that true AP and lateral imaging are especially important determinants of the true relationship between the electrode tip and SAP, as visualized under fluoroscopy.

## Declaration of competing interest

The authors declare that they have no known competing financial interests or personal relationships that could have appeared to influence the work reported in this paper.
